# Life’s Essential 8 in relation to self-rated health and health-related quality of life in a large population-based sample: the SCAPIS project

**DOI:** 10.1007/s11136-023-03580-1

**Published:** 2024-01-25

**Authors:** Ángel Herraiz-Adillo, Viktor H. Ahlqvist, Bledar Daka, Josefin Wångdahl, Patrik Wennberg, Jakob Carlsson, Sara Higueras-Fresnillo, Cecilia Lenander, Carl Johan Östgren, Daniel Berglind, Karin Rådholm, Pontus Henriksson

**Affiliations:** 1 Department of Health, Medicine and Caring Sciences, Linköping University, Linköping, Sweden; 2 Department of Global Public Health, Karolinska Institutet, Stockholm, Sweden; 3 School of Public Health and Community Medicine, Institute of Medicine, Sahlgrenska Academy, University of Gothenburg, Gothenburg, Sweden; 4 Aging Research Center, Karolinska Institutet & Stockholm University, Stockholm, Sweden; 5 Department of Public Health & Caring Sciences, Uppsala University, Uppsala, Sweden; 6 Department of Public Health and Clinical Medicine, Family Medicine, Umeå University, Umeå, Sweden; 7 Department of Preventive Medicine and Public Health, Universidad Autónoma de Madrid, Madrid, Spain; 8 Department of Clinical Sciences in Malmö, Centre for Primary Health Care Research, Lund University, Lund, Sweden; 9 Centre of Medical Image Science and Visualization (CMIV), Linköping University, Linköping, Sweden; 10 Centre for Epidemiology and Community Medicine, Region Stockholm, Stockholm, Sweden; 11 The George Institute for Global Health, University of New South Wales, Sydney, Australia

**Keywords:** Health-related quality of life, Ideal cardiovascular health, Life's Essential 8, Quality of life, Self-rated health

## Abstract

**Purpose:**

To monitor cardiovascular health, in 2022, the American Heart Association (AHA) updated the construct “Life’s Simple 7” (LS7) to “Life’s Essential 8” (LE8). This study aims to analyze the associations and capacity of discrimination of LE8 and LS7 in relation to self-rated health (SRH) and health-related quality of life (HRQoL).

**Methods:**

This study from the Swedish CArdioPulmonary bioImage Study (SCAPIS) included 28 731 Swedish participants, aged 50–64 years. Three different scores were derived from the SF-12 questionnaire: 1-item question SRH (“In general, would you say your health is …?”), mental-HRQoL and physical-HRQoL. Logistic regression, restricted cubic splines, and ROC analysis were used to study the associations between the AHA scores in relation to SRH and HRQoL.

**Results:**

Compared to those with a LE8 score of 80, participants with a LE8 score of 40 were 14.8 times more likely to report poor SRH (OR: 14.8, 95% CI: 13.0–17.0), after adjustments. Moreover, they were more likely to report a poor mental-HRQoL (OR: 4.9, 95% CI: 4.2–5.6) and a poor physical-HRQoL (OR: 8.0, 95% CI: 7.0–9.3). Area under curves for discriminating poor SRH were 0.696 (95% CI: 0.687–0.704), 0.666 (95% CI: 0.657–0.674), and 0.643 (95% CI: 0.634–0.651) for LE8, LS7 (0–14), and LS7 (0–7), respectively, all p values < 0.001 in the DeLong’s tests.

**Conclusion:**

LE8 and LS7 had strong and inverse associations with SRH, mental-HRQoL, and physical-HRQoL, though LE8 had a somewhat higher capacity of discrimination than LS7. The novel LE8, a construct initially conceived to monitor cardiovascular health, also conveys SRH and HRQoL.

**Supplementary Information:**

The online version contains supplementary material available at 10.1007/s11136-023-03580-1.

## Plain English Summary

Cardiovascular diseases are a significant health concern, making it crucial to monitor cardiovascular health. To overcome the limitations of Life's Simple 7 (LS7) in monitoring cardiovascular health, in June 2022, the American Heart Association introduced a new indicator: the Life's Essential 8 (LE8). Understanding the relationship between cardiovascular health and both self-rated health and health-related quality of life can provide valuable insights for improving overall well-being. Thus, this study explores how LE8 and LS7 are related to self-rated health and health-related quality of life (including its mental and physical components). We analyzed data from a large middle-aged population (28 731 Swedish participants) with comprehensive measurements of LE8, LS7, self-rated health, and health-related quality of life. Our findings revealed that participants with lower LE8 scores were much more likely to report poor self-rated health, as well as poor mental and physical scores in health-related quality of life. Furthermore, LE8 demonstrated a slightly better ability to distinguish individuals with poor self-rated health and poor health-related quality of life compared to LS7. In conclusion, the novel LE8, an indicator initially conceived to monitor cardiovascular health, also conveys self-rated health and health-related quality of life.

## Introduction

Positive trends in cardiovascular mortality in the United States and Europe over the last few decades have reverse or stalled after 2010 [1, 2]. Consequently, cardiovascular disease continues to be a major cause of mortality and morbidity worldwide, with 17.8 million deaths in 2017, accounting for 31.8% of all global deaths [3]. The increase in population levels of cardiovascular risk factors (e.g., obesity and diabetes) has played a significant role in this reversed trend in the burden of cardiovascular disease [1, 2], highlighting the importance of primary prevention.

To mitigate the burden of cardiovascular disease, in 2010, the American Heart Association (AHA) defined the construct “ideal cardiovascular health” or “Life’s Simple 7” (LS7) [4]. It focused not only on primary but also on primordial prevention (preventing the development of risk factors), thus representing a crucial shift from cardiovascular disease management to population-based cardiovascular health promotion. In June 2022, a revised construct was defined by the AHA, the “Life’s Essential 8” (LE8) [5]. Compared to LS7, LE8 includes a new component (sleep health) and revised calculations of previous behavior (diet, physical activity, body mass index [BMI], and nicotine exposure) and factor scores (non-high-density lipoprotein [HDL] cholesterol, glucose/glycosylated hemoglobin, and blood pressure). Thus, LE8 defines an integral definition of health, based both on health factors and health behaviors.

Aligned with a holistic understanding of health, there is a growing emphasis on the significance of self-rated health (SRH) and health-related quality of life (HRQoL). SRH is a subjective indicator of health status that integrates biological, mental, social, and functional aspects of a person, including individual and cultural beliefs and health behaviors [6]. Furthermore, the HRQoL is usually described as: “A term referring to the health aspects of quality of life, generally considered to reflect the impact of disease and treatment on disability and daily functioning” or as “a term that reflects the impact of perceived health on an individual’s ability to live a fulfilling life”[7].

In the literature, there is abundant evidence that good SRH and HRQoL are associated with healthier cardiovascular risk profiles [8, 9], lower incidence of fatal and non-fatal cardiovascular events [10–12], lower all-cause mortality [13–16] and less healthcare utilization [16, 17]. Similarly, several studies have confirmed the association of LS7 and cardiovascular disease and mortality [18, 19]. Although there are currently studies showing that LE8 is strongly linked to the atherosclerotic burden [20], cardiovascular disease [21, 22], as well as cardiovascular and all-cause mortality [23, 24], no studies have examined LE8 in relation to SRH or HRQoL. Whether the utility of the novel LE8 score extends beyond its intended purpose to monitor cardiovascular health is important, since routine collection of LE8 in cardiovascular medicine could be used to convey other aspects of health including SRH or HRQoL.

Thus, this study aims i) to analyze the cross-sectional associations between LE8 and LS7 in relation to SRH and HRQoL (measured as SRH, mental-HRQoL, and physical-HRQoL) and ii) to compare the capacity for discriminating poor SRH, mental-HRQoL, and physical-HRQoL between LE8 and LS7 scores.

## Materials and methods

### Study design and participants

This population-based study used data from the Swedish CArdioPulmonary bioImage Study (SCAPIS), which protocol has been previously described in detail [25]. During 2013–2018, SCAPIS randomly selected a large population (*n* = 30 154, overall participation rate = 50.3%) located at 6 university sites in Sweden (Linköping, Malmö/Lund, Stockholm, Umeå, Göteborg, Uppsala) to study prevention strategies for cardiovascular disease.

Supplementary Fig. 1 depicts the flow chart for the study. Out of the 30 154 participants available in SCAPIS, 28 971 (96.1%) reported their SRH. Of those, after excluding participants with missing data to calculate at least 7 components in LE8 and LS7 scores, 28 731 (95.3%) and 25 714 (85.3%) participants were retained and used in the analysis of LE8 and LS7, respectively.

The Swedish Ethical Review Authority granted ethical approval (reference numbers: 2021–06408-01, 2022–04375-02), and all participants provided written informed consent to participate in the study.

### Study variables

#### Life’s Essential 8

LE8 was defined based on the AHA criteria and incorporates 4 health behaviors: diet, physical activity, nicotine exposure, and sleep health, and 4 health factors measurements: BMI, non-HDL cholesterol, fasting blood glucose/glycosylated hemoglobin, and blood pressure [5]. Details about measurement and calculation of health behaviors and health factors in LE8 and LS7 have been published elsewhere [20].

In brief, regarding health behaviors, dietary habits were evaluated using the web-based questionnaire (MiniMeal-Q) and the scores were adapted from the Mediterranean Eating Pattern for Americans (MEPA) [26]. Physical activity was measured over a 7 days period with three different tri-axial accelerometers: Actigraph GT3X + , wGT3X +,  and wGT3X-BT (ActiGraph LCC, Pensacola, FL, USA) [27], considering ≥ 2690 counts per minute as moderate-vigorous intensity physical activity [28]. Nicotine exposure and sleep health were assessed with self-administered questionnaires. For health factors, standardized laboratory and clinical procedures were used, and medication consumption was collected from the Swedish Prescribed Drug Register.

To calculate an overall LE8 score, all 8 components within LE8 were scored from 0 to 100 (0, the worst health; 100, the best health). Following the AHA recommendations, an overall LE8 score was calculated as the unweighted average of all present components (range: 0–100). A minimum of 7 components was required for computing the overall LE8 score. In addition, two different scores, ranging from 0 to 100, were calculated for LE8 behaviors and LE8 factors as the unweighted average of all present components in behaviors and factors, respectively. In both cases, a minimum of 3 reported components was required for computing behaviors and factors scores.

#### Life’s Simple 7

LS7 was defined in accordance with the AHA criteria and incorporates 4 health behaviors: diet, physical activity, BMI, and smoking status, and 3 health factors: total cholesterol, blood glucose, and blood pressure [4]. In SCAPIS, with the exception of sleep health, the measurements, techniques, and questionnaires to obtain the LS7 score were similar to those in LE8, though the calculation of scores differed [20]. In LS7, as the AHA recommended, dietary habits were consistent with the Dietary Approaches to Stop Hypertension (DASH) eating plan [4].

To compute the LS7 score, all 7 components were required for computation, and two scoring algorithms were created. In the LS7 (0–7) score, the total score is equal to the number of components at the ideal level, with 0 representing the worst health and 7 representing the best health status. The LS7 (0–14) score is calculated as the sum of components scored as “poor = 0,” “intermediate = 1,” and “ideal = 2 points,” yielding a total score from 0 to 14, with 0 representing the worst health and 14 representing the best health status. In addition, two different scores were calculated for LS7 behaviors and LS7 factors as the sum of all present components in behaviors and factors, respectively. All 4 behaviors and all 3 factors were necessary to compute behaviors and factors scores, respectively.

#### Self-rated health and health-related quality of life

In SCAPIS, from the Swedish SF-12.V1 questionnaire, which is an adaptation of the Swedish SF-36 questionnaire [29–31], three different scores were derived to operationalize SRH and HRQoL. The construct SRH was measured with the 1-item question “In general, would you say your health is …?”, which was scored on a 5-point Likert scale as: poor, fair, good, very good, and excellent. Despite its simplicity, this 1-item self-reported question (hereafter referred to as “SRH”) has proved good validity to measure “general health,” adequately integrating the physical, psychological, and social dimensions in health [6]. In accordance with previous publications [32–34], the SRH score was dichotomized into poor (poor and fair) and good (good, very good or excellent).

By contrast, HRQoL is a multidimensional index referring to the physical, psychological, and social domains of health and well-being [7]. The Swedish SF-12.V1 questionnaire aggregates the eight subscales of SF-36 allowing for the calculation of two different summary scores: “mental-HRQoL” and “physical-HRQoL” [35]. Both scores were computed following a modified protocol based on Ware’s framework [35], utilizing Farivar’s weights [36] instead of Ware’s original weights. We did not use Ware’s orthogonally rotated weights, as they force mental and physical scores to be uncorrelated, despite evidence of their non-independence [37]. Thus, the validity and interpretation of the orthogonal approach have been criticized [36–39]. Instead, we used weights derived from oblique (correlated) factor analysis as Farivar et al. proposed [36]. In brief, we initially reversed the SCAPIS SF-12.V1 scores to ensure higher values consistently indicated better health. We then utilized dummy variables to categorize item responses, and later multiplied scores in dummy variables by suggested weights, incorporating a constant.

In the Swedish SF-12.V1 questionnaire, questions 9 to 12 (9, “Feelings last 4 weeks: felt calm and peaceful”; 10, “Feelings last 4 weeks: had a lot of energy”; 11, “Feelings last 4 weeks: felt downhearted and blue”; and 12, “Health limitations, last 4 weeks: interference with social activities”) only had five response options (0, “All of the time”; 1, “Most of the time”; 2, “Some of the time”; 3, “A little of the time”; 4, None of the time”) instead of six as in the original Ware’s manual [35]. In this manual, 5 weights (response options minus 1) were considered to score the 6-items 9–12 questions. In our work, we assigned Farivar’s weights for first (1) and last category (5) to our first (1) and last category (4), and then we linearly distributed the difference between first and last categories into the second and third categories [36]. The cutoffs for poor mental-HRQoL and poor physical-HRQoL were defined as the sample mean minus 1 standard deviation.

#### Statistical analysis

We conducted a complete case analysis, excluding participants with incomplete data on exposures, outcomes, and main covariates in the main model (i.e., Model 2). Descriptive statistics are presented as means and standard deviations for the continuous variables or frequencies and percentages for the categorical variables.

**Fig. 1:**
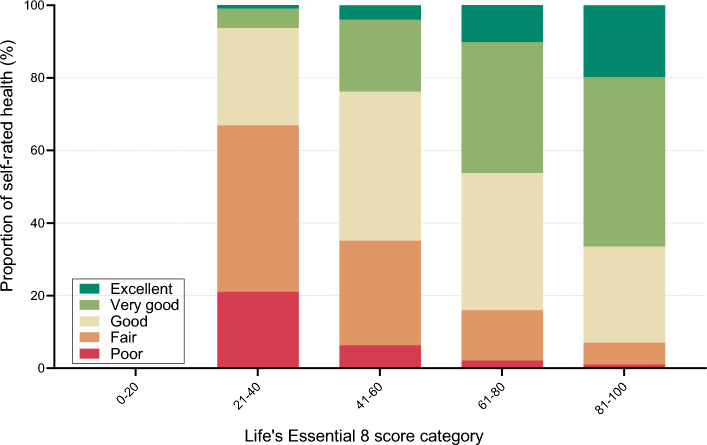
Distribution of the levels of self-rated health along Life’s Essential 8 categories

**Fig. 2:**
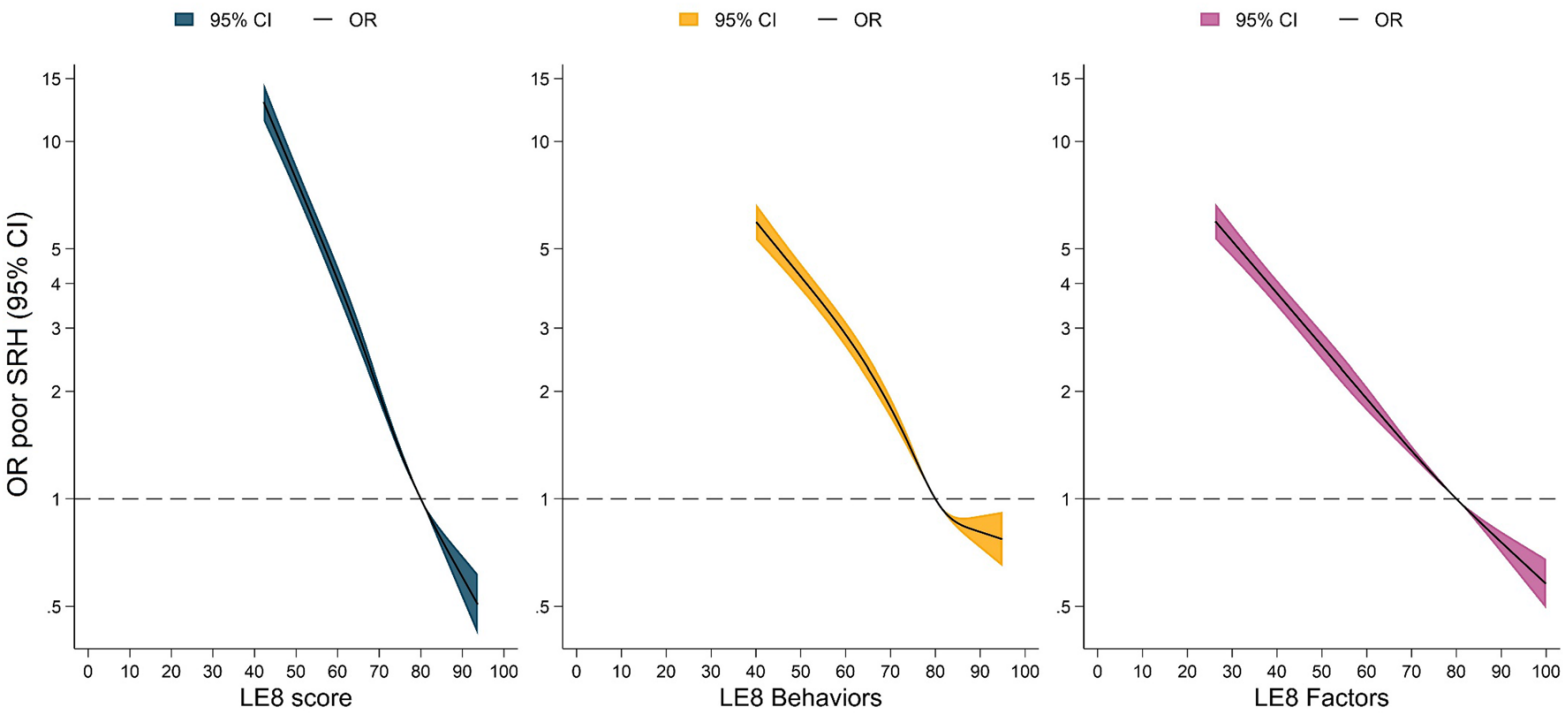
Restricted cubic splines for the association of Life’s Essential 8, Life’s Essential 8 behavior, and Life’s Essential 8 factor scores with poor self-rated health. All models are binary logistic regressions adjusted by age, sex, and site. X-axes were trimmed to depict the associations for the 1st to 99th percentile of LE8 values. Reference points are settled at 80 points, which represent the 78.2th, 58.7th, and 76.1th percentiles for LE8, LE8 behaviors, and LE8 factors, respectively. *CI* confidence interval, *OR* odds ratio, *LE8* Life’s Essential 8 score, *SRH* self-rated health

First, we examined the distribution of the levels of self-rated health along Life´s Essential 8 categories (Fig. 1). Second, we examined associations of LE8 and LS7 with SRH through binary logistic regression models since SRH is typically characterized on a dichotomous scale (i.e., excellent/very good/good vs fair/poor) (Fig. 2). To allow for potential non-linear associations, we modeled the associations using restricted cubic splines with 4 knots [40] located at percentiles 5th, 35th, 65th, and 95th across LE8 and LS7, respectively. Reference values in LE8, LE8 behaviors, and LE8 factors were settled at 80 points, which aligns with the cut-off proposed by the AHA to define high cardiovascular health [5], see corresponding percentiles in Fig. 2. For LS7, to maximize the distributional comparability to the 80-point cut-off in LE8, the references were settled at 4 and 10 points for LS7 (0–7) and LS7 (0–14), respectively. Similarly, reference for LS7 behaviors and factors were settled at the point that render the most similar distribution to the global LS7 score, see corresponding percentiles in Supplementary Fig. 4 and 6. To test whether the association between poor SRH, poor mental-HRQoL and poor physical-HRQoL differed by sex in relation to LE8 and LS7 scores, a multiplicative interaction term was included in the models. Models were calculated considering 3 increasing levels of covariate adjustment: Model 1, unadjusted; Model 2, adjusted by sex, age, and study site (Linköping, Stockholm, Gothenburg, Lund/Malmö, Uppsala, Umeå); Model 3, adjusted by Model 2 covariates + educational status, marital status, and chronic disease (myocardial infarction, stroke, heart failure, peripheral arterial disease, chronic obstructive pulmonary disease, celiac disease, Crohn’s disease or ulcerative colitis disease, rheumatic disease, and cancer). Model 2 was considered as the main statistical model considering its clinical utility since it includes important covariates easy to collect in clinical practice while remaining relatively straightforward and easy to interpret.

**Fig. 3:**
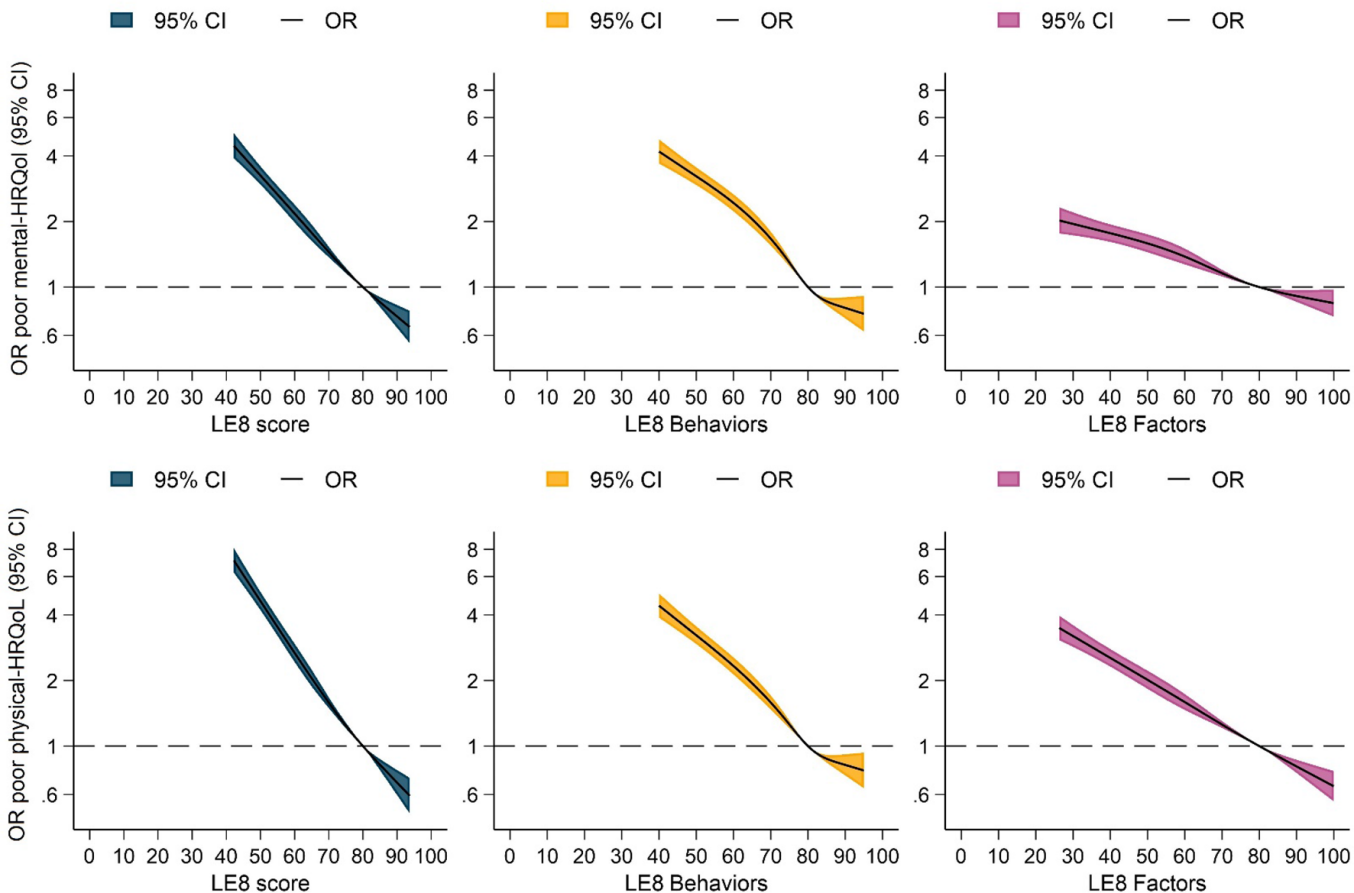
Restricted cubic splines for the association of Life’s Essential 8, Life’s Essential 8 behavior, and Life’s Essential 8 factor scores with poor mental health-related quality of life (upper row) and poor physical health-related quality of life (lower row). All models are binary logistic regressions adjusted by age, sex, and site. Reference points are settled at 80 points. X-axes were trimmed to depict the associations for the 1st to 99th percentile of LE8 values. *CI* confidence interval, *OR* odds ratio, *LE8* Life’s Essential 8 score, *HRQoL* health-related quality of life

Third, we further examined associations of LE8 and LS7 with poor mental-HRQoL and physical-HRQoL using Model 2 and similar reference points as described above (Fig. 3).

**Fig. 4:**
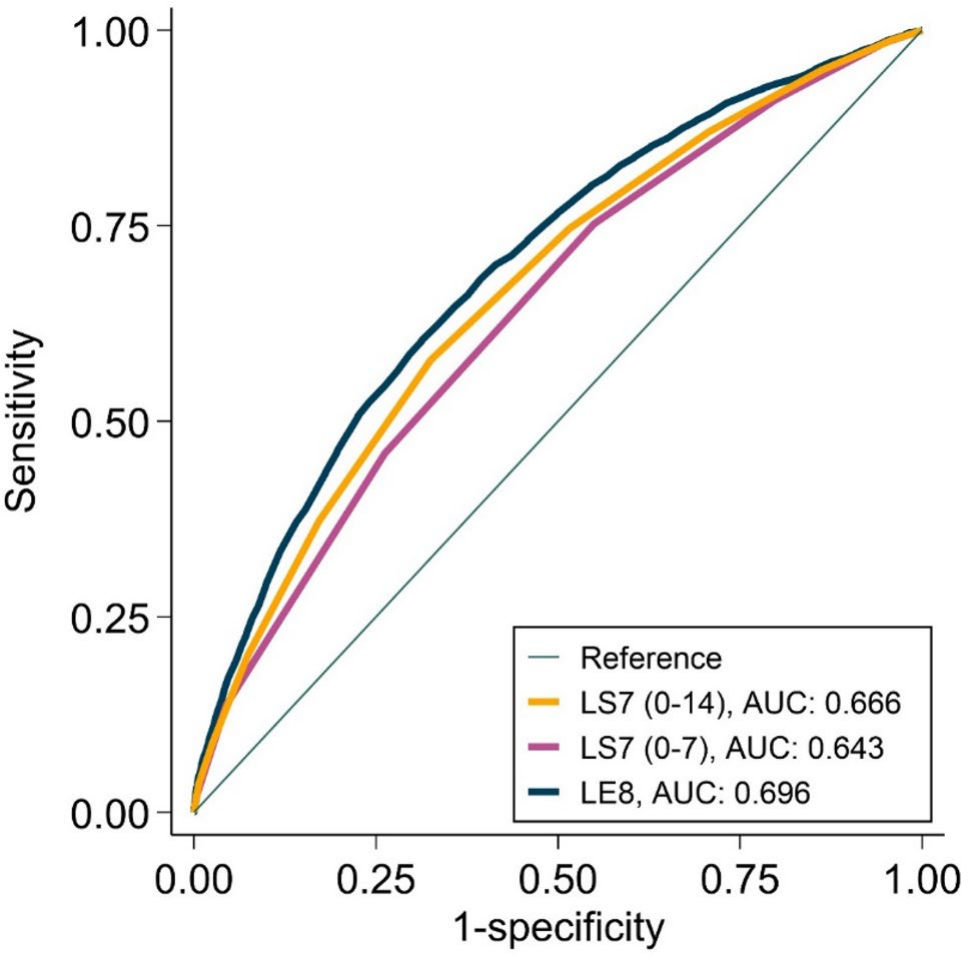
Receiver operating characteristic curves of Life’s Essential 8, Life’s Simple 7 (0–7), and Life’s Simple 7 (0–14) to discriminate poor self-rated health. *AUC* area under curve, *LE8* Life’s Essential 8 score, *LS7 (0–7)* Life’s Simple 7 (scored as 0 to 7), *LS7 (0–14)* Life’s Simple 7 (scored as 0 to 14)

Fourth, we analyzed the capacity to discriminate poor SRH, poor mental-HRQoL and poor physical-HRQoL between LE8, LS7 (0–7) and LS7 (0–14) scores through receiver operating characteristic (ROC) curves and areas under the ROC curves (AUC) were compared with DeLong´s tests (Fig. 4).

Fifth, the associations of the different components of the LE8 score with SRH were studied through logistic regression models considering components as continuous variables (0–100) transformed to Z scores (Supplementary Fig. 3).

Finally, to examine the robustness of our main findings, we conducted a sensitivity analysis for the associations of LE8 and SRH by only including those with complete data on all LE8 components (instead of including those with data on ≥ 7 components).

Two-tailed p values < 0.05 were considered significant in all analyses and statistical analysis were performed using IBM SPSS Statistics Program (version 28.0, IBM Corp., NY, USA) and Stata 17 (StataCorp. 2021).

## Results

### Descriptive statistics

After exclusions, 28 731 (95.3%) participants were considered for the main analysis (mean age, 57.5 years; 51.5% female). Table 1 summarizes the demographic and clinical characteristics of the study population by sex. In general, participants excluded from the study had poorer cardiovascular health, lower levels of university education achievement, and lower SRH and HRQoL compared to included participants (Supplementary Table 1).

**Table 1:** Demographic and clinical characteristics of the study population by sex

	Total *n* = 28 731	Women *n* = 14 809 (51.5%)	Men *n* = 13 922 (48.5%)
Age and cardiovascular risk factors			
Age, y	57.5 ± 4.3	57.5 ± 4.3	57.5 ± 4.4
BMI, kg/m^2^	26.9 ± 4.4	26.5 ± 4.8	27.4 ± 3.9
Obesity, (BMI ≥ 30 kg/m^2^)	6082 (21.2)	3014 (20.4)	3068 (22.0)
Total cholesterol, mg/dL	212.2 ± 40.6	218.1 ± 39.4	205.8 ± 40.8
Hypercholesterolemia	3342 (11.8)	1396 (9.5)	1946 (14.2)
Systolic blood pressure, mmHg	125.8 ± 17.0	123.1 ± 17.7	128.8 ± 15.6
Diastolic blood pressure, mmHg	77.5 ± 10.5	76.6 ± 10.8	78.5 ± 10.1
Hypertension	6450 (22.7)	3069 (21.0)	3381 (24.6)
Fasting glucose, mg/dL	103.2 ± 19.8	99.9 ± 17.1	106.7 ± 21.7
Glycosylated hemoglobin, mmol/mol	36.5 ± 6.3	36.2 ± 5.5	36.8 ± 7.1
Diabetes mellitus	1235 (4.4)	452 (3.1)	783 (5.7)
Moderate-vigorous physical activity, min/week	391.7 ± 208.4	378.6 ± 195.9	405.7 ± 220.1
LE8 diet (0–100) score	41.1 ± 16.1	44.6 ± 16.2	37.3 ± 15.0
Smoking			
Current	3571 (12.6)	1849 (12.6)	1722 (12.5)
Ex-smoker ≤ 1 year	420 (1.5)	230 (1.6)	190 (1.4)
Ex-smoker > 1 year	9962 (35.0)	5487 (37.5)	4475 (32.4)
Never	14,484 (50.9)	7080 (48.3)	7404 (53.7)
Education level			
Unfinished primary school	177 (0.6)	86 (0.6)	91 (0.7)
Primary school	2444 (8.5)	1095 (7.4)	1349 (9.7)
Secondary school	13 029 (45.5)	6285 (42.6)	6744 (48.6)
University degree	12 987 (45.4)	7299 (49.4)	5688 (41.0)
Financial strain^**1**^			
Yes	1528 (5.3)	833 (5.6)	695 (5.0)
No	27 019 (94.1)	13 894 (93.8)	13 135 (94.3)
Current marital status			
Single	3822 (13.4)	2041 (13.9)	1781 (12.9)
Divorced	3161 (11.1)	2037 (13.8)	1124 (8.1)
Married	21 107 (73.9)	10 292 (69.9)	10 815 (78.1)
Widow	475 (1.7)	356 (2.4)	119 (0.9)
Birth country			
Sweden	24 112 (84.3)	12 372 (83.9)	11 740 (84.7)
Other country	4498 (15.7)	2381 (16.1)	2117 (15.3)
Cardiovascular health scores			
LE8 (0–100)	70.7 ± 11.6	72.7 ± 11.7	68.6 ± 11.1
LS7 (0–7)	3.3 ± 1.3	3.5 ± 1.3	3.0 ± 1.2
LS7 (0–14)	9.1 ± 2.0	9.5 ± 2.0	8.8 ± 1.9
Self-rated health			
Poor	801 (2.8)	469 (3.2)	332 (2.4)
Fair	4367 (15.2)	2353 (15.9)	2014 (14.5)
Good	10 340 (36.0)	5098 (34.4)	5242 (37.7)
Very good	10 044 (35.0)	5359 (36.2)	4685 (33.7)
Excellent	3179 (11.1)	1530 (10.3)	1649 (11.8)
Health-related quality of life			
Poor mental health	4190 (15.1)	2622 (18.3)	1568 (11.6)
Poor physical health	4159 (15.0)	2620 (18.3)	1539 (11.4)

Higher LE8 and LS7 scores indicate better cardiovascular health

Data refer to mean ± standard deviation and frequencies (percentage)

*BMI* body mass index, *LE8* Life’s Essential 8 score, *LS7 (0–7)* Life’s Simple 7 (scored as 0–7), *LS7 (0–14)* Life’s Simple 7 (scored as 0–14)

^1^Difficulties in managing regular expenses in the last 12 months

Regarding cardiovascular health, the mean for LE8, LS7 (0–7), and LS7 (0–14) scores were 70.7, 3.3, and 9.1 points, respectively. In terms of SRH, 18.0% of participants reported poor scores (including poor and fair responses), while 15.1% and 15.0% had poor scores for mental-HRQoL and physical-HRQoL, respectively. Despite women had higher percentages of poor scores in SRH and HRQoL compared to men, women exhibited better scores in cardiovascular health (72.7 vs 68.6 points in LE8 for women and men, respectively). Specifically, women had less obesity, hypercholesterolemia, high blood pressure, and diabetes than men, but lower levels of physical activity.

### Life’s Essential 8 in relation to self-rated health

The overall and segregated by sex distribution of the prevalences for SRH along different groups of LE8 can be seen in Fig. 1 and Supplementary Fig. 2. The association between LE8 and SRH did not vary by sex (p value for interaction in all models > 0.05).

Figure 2 depicts the restricted cubic splines of poor SRH with LE8, showing strong and inverse associations throughout the entire range of LE8. When adjusted for age, sex, and site, a score of 40 points in LE8 was associated with roughly fifteen times higher odds ratio (OR) (14.8, 95% CI: 13.0–17.0) compared to the reference group (80 points), detailed data in Supplementary Table 2. Similarly, strong, and inverse trends were observed in both LE8 health behaviors and health factors, although health behaviors showed somewhat stronger associations with SRH than factors. All eight components were found to have significant associations with poor SRH, ranging from ORs of 0.57 (95% CI: 0.55–0.58) for BMI to 0.87 (95% CI: 0.84–0.90) for diet (Supplementary Fig. 3).

In a sensitivity analysis, only analyzing those with complete data for all 8 LE8 components (instead of those with data on ≥ 7 components) did not significantly change the results (Supplementary Table 3).

### Life’s Essential 8 in relation to mental and physical health-related quality of life

LE8 also had strong and inverse associations with both mental-HRQoL and physical-HRQoL scores (Fig. 3**)**. Specifically, after adjusting for age, sex, and site, a score of 40 points in LE8 was associated with approximately five (OR: 4.9, 95% CI: 4.2–5.6) and eight (OR: 8.0, 95% CI: 7.0–9.3) higher odds of poor mental-HRQoL and poor physical-HRQoL, respectively, compared to the reference group (80 points). Moreover, both LE8 behaviors and LE8 factors were strongly associated with mental-HRQoL and physical-HRQoL (Supplementary Table 4).

### Life’s Simple 7 in relation to self-rated health, and mental and physical health-related quality of life

Supplementary Fig. 4 depicts the restricted cubic splines of LS7 (0–7) with poor SRH, showing strong and inverse associations. Thus, when adjusted for age, sex, and site, a score of 1 point in LS7 (0–7) was associated with nearly five times the odds (OR: 4.6, 95% CI: 4.1–5.1) of poor SRH compared to the reference group (4 points). In consonance with LE8, behaviors seemed to have somewhat stronger associations than factors. In accordance with LE8, LS7 (0–7) also showed strong and inverse associations with mental-HRQoL and physical-HRQoL (Supplementary Fig. 5).

Supplementary Fig. 6 and 7 depict the restricted cubic splines of LS7 (0–14) with SRH and with mental-HRQoL and physical-HRQoL, respectively. Overall, similar patterns were observed in LS7 (0–14) compared to LS7 (0–7).

### Life’s Essential 8 vs Life’s Simple 7

LE8 had a slightly better ability to distinguish individuals with poor SRH (AUC: 0.696, 95% CI: 0.687–0.704) compared to LS7 (0–14) (AUC: 0.666, 95% CI: 0.657–0.674) and LS7 (0–7) (AUC: 0.643, 95% CI: 0.634–0.651), see Fig. 4. The DeLong’s tests showed that AUC for LE8 was significantly larger than AUC for LE7 (0–7) (*p* < 0.001) and for LS7 (0–14) (*p* < 0.001).

Regarding mental-HRQoL, AUCs were 0.592 (95% CI: 0.582–0.602), 0.560 (95% CI: 0.550–0.570), and 0.547 (95% CI: 0.537–0.557) for LE8, LS7 (0–14) and LS7 (0–7), respectively, all p values < 0.001 in the comparison of AUCs. Finally, regarding physical-HRQoL, AUCs were 0.635 (95% CI: 0.625–0.645), 0.605 (95% CI: 0.595–0.615), and 0.591 (95% CI: 0.581–0.601) for LE8, LS7 (0–14) and LS7 (0–7), respectively, all p values < 0.001 in the comparison of AUCs (Supplementary Fig. 8).

## Discussion

This large population-based study of middle-aged participants from SCAPIS provides evidence for graded associations of the new LE8 score with SRH, mental-HRQoL, and physical-HRQoL. These associations remained robust after adjusting for sociodemographic factors and chronic diseases. In addition, the novel LE8 score performed slightly better than the conventional LS7 in discriminating SRH and HRQoL, regardless of whether LS7 was scored as 0–7 or 0–14.

Though several studies have examined the association of LS7 in relation to SRH and HRQoL [32–34, 41, 42], our study is, to the best of our knowledge, the first to investigate the association between LE8 in relation to SRH and HRQoL, as well as to compare the discrimination capacity of LE8 vs LS7. Thus, our study expands upon prior research by demonstrating that LE8, a novel construct originally designed by the AHA to monitor cardiovascular health, also conveys SRH and the mental and physical components of HRQoL.

In our study, poor SRH was almost 15-fold higher among participants with a LE8 score of 40 points compared to those with a score of 80 points. Similarly, in LS7, when scored as the sum of ideal components (0–7), poor SRH was almost 5-fold higher among participants with only 1 point in LS7 compared to those with a score of 4 points. While differences in the categorization of LS7, SRH, and HRQoL, as well as variations in sociodemographic profiles across populations, may limit direct comparisons with previous studies, our findings seem generally consistent with those reported in prior cross-sectional studies. For example, the Multi-Ethnic Study of Atherosclerosis found that compared to the poor-fair group, those with excellent and very good SRH had ORs of 4.9 and 2.2 for optimal cardiovascular health (defined as 11–14 in the LS7 (0–14) score), respectively [34]. Similar findings were reported in the National Health and Nutrition Examination Survey [32]. Noteworthy, even higher associations were observed in specific populations, such as healthy employees in the Baptist Health South Florida study [33]. Not surprisingly, a better knowledge and self-awareness of the cardiovascular health status may strengthen the association between cardiovascular health and SRH or HRQoL. Interestingly, these aforementioned positive associations were also observed in a longitudinal study [41].

In June 2022, the AHA launched the new construct LE8 to overcome some of the LS7 limitations, particularly the restricted sensitivity in measuring inter-individual variation and intra-individual changes over time [5]. In contrast to previous findings in SCAPIS, which reported similar predictive capacity for coronary stenosis and carotid plaques between LE8 and LS7 [20], in the present study, LE8 performed slightly better than LS7 for discriminating a poor outcome in SRH, mental-HRQoL, and physical-HRQoL. Though the clinical utility of this improvement is probably small, the updated scoring algorithm and the inclusion of sleep health in LE8 seem to have some added value beyond LS7 to convey SRH and HRQoL. In fact, in a separate analysis of the different components in LE8, sleep health showed one of the strongest associations with SRH, which is in consonance with another study [43], and could partially explain the improvement in the discrimination capacity exhibited by LE8. This fact would be in favor of the proposed change by the AHA from LS7 to LE8, and emphasizes an integral concept of health, considering different cardiovascular health components as latent components of SRH and HRQoL (and vice versa).

In general, with the exception of tobacco, evidence supports a stronger association of health factors vs behaviors with cardiovascular outcomes [44]. However, the relationship between health factors vs behaviors and both SRH and HRQoL could be different, as it is complex and multifaceted. For instance, Veromaa et al. found a stronger association between LS7 health behaviors and SRH [42], although this pattern has not been consistently replicated by others [33, 34]. In our study, after adjustments, both LE8 health factors and health behaviors were found to be significantly associated with SRH, with the strongest associations observed for BMI and sleep health. In this sense, the association of SRH and LE8 behaviors may be bidirectional. On the one hand, behaviors could have positive effects not only on cardiovascular health but also on general health and well-being, including improved mental health and a better quality of life. On the other hand, people with positive psychological well-being seem to be more prone to engage in healthy behaviors such as higher physical activity, smoking abstinence or healthier diet [45–47]. Additionally, individuals may have higher self-awareness for behaviors than factors, especially in populations with low health coverage or low health literacy, where factors could be unnoticed or underemphasized. Finally, an interesting hypothesis is that SRH seems to have a biologic basis, involving latent physiological variables that could be a sensitive barometer of the physiologic status [48].

This study has several strengths. Firstly, it included a large randomly selected population, representing over 95% of the SCAPIS population with minimal missing data. Secondly, SCAPIS conducted comprehensive clinical examinations, enabling the computation of complete cardiovascular health scores that meet the AHA criteria [4, 5]. Specifically, SCAPIS measured physical activity over 7 days using tri-axial accelerometers, instead of relying on self-reported questionnaires. Finally, our study delved into the analysis of HRQoL by also evaluating its mental and physical components.

Limitations of the study should be considered. Firstly, the relatively narrow range of age of the participants (50–64 years) limits generalizability of the results to other populations. Secondly, SCAPIS refers to a Swedish population, so the generalization to other regions may be compromised. Furthermore, 15.7% of participants in this study were born outside Sweden, which is somewhat lower than the average of immigrants (around 20%) in the 50–64 years age group in Sweden [49]. This is an important factor to consider since ethnicity has been linked to differences in SRH and HRQoL [50]. Thirdly, despite a low percentage of missing data and in line with findings from other studies both outside [51, 52] and within SCAPIS [53, 54], low socio-economic areas were underrepresented. Thus, excluded participants in the study exhibited a worse cardiovascular risk profile, which raises concern for some selection bias and healthy volunteer effect. Nevertheless, this probably has underestimated the true association of LE8 and LS7 with SRH and HRQoL. Finally, to compute mental and physical components of HRQoL, we used the standard United Stated (U.S.) weights in the scoring algorithms instead of the Swedish ones [36]. Nevertheless, there is empirical evidence of little difference between the use of these different scores and some authors recommend using U.S. weights for better comparison and interpretation across countries in relation to U.S. standard benchmarks [55].

## Conclusions and clinical implications

In conclusion, this study provides evidence of significant associations between the AHA cardiovascular health scores and SRH, as well as the mental and physical components of HRQoL. Additionally, the LE8 score demonstrates slightly superior capacity for discriminating poor SRH and HRQoL outcomes than the LS7 score. Therefore, the LE8, a novel score originally designed to monitor cardiovascular health, appears also to convey SRH and HRQoL. Overall, these findings, in consonance with a holistic approach of health, highlight the potential utility of the LE8 score as a comprehensive and integral indicator of cardiovascular health and both SRH and HRQoL. Awareness of patients with low LE8 and LS7 scores is warranted, since this may indicate a poor SRH and HRQoL, which have been related not only with cardiovascular, but also with non-cardiovascular poor outcomes and more healthcare utilization. Nevertheless, further work including longitudinal studies are needed to corroborate our novel associations of LE8 scores with SRH and HRQoL.

### Supplementary Information

Below is the link to the electronic supplementary material.

Supplementary file1 (DOCX 1230 kb)

## Data Availability

The data underlying this article cannot be shared publicly due to legal reasons as well as the privacy of individuals that participated in the study. However, by contacting the study organization (www.scapis.org) or corresponding author, information will be provided regarding the procedures for accessing data following Swedish legislation.
