# Supplementary material for: Life’s Essential 8 in relation to self-rated health and health-related quality of life in a large population-based sample: the SCAPIS project
Source: Qual Life Res. 2024 Jan 25;33(4):1003–14. doi: 10.1007/s11136-023-03580-1 (PMC10973036; doi:10.1007/s11136-023-03580-1)
Supplement: Supplementary file 1 — Supplementary file1 (DOCX 1230 kb) [file 11136_2023_3580_MOESM1_ESM.docx]

**Supplementary material**

**Life’s Essential 8 in relation to**

**self-rated health and health-related quality of life**

**in a large population-based sample: the SCAPIS project**

**Ángel Herraiz-Adillo^1^, Viktor H. Ahlqvist^2^, Bledar Daka^3^, Josefin Wångdahl^4,5^, Patrik Wennberg^6^, Jakob Carlsson^1^, Sara Higueras-Fresnillo^1,7^, Cecilia Lenander^8^, Carl Johan Östgren^1,9^, Daniel Berglind^2,10^, Karin Rådholm^1,11^, Pontus Henriksson^1^.**

1. Department of Health, Medicine and Caring Sciences, Linköping University, Linköping, Sweden.

2. Department of Global Public Health, Karolinska Institutet, Stockholm, Sweden.

3. School of Public Health and Community Medicine, Institute of Medicine, Sahlgrenska Academy, University of Gothenburg, Gothenburg, Sweden.

4. Aging Research Center, Karolinska Institutet & Stockholm University, Stockholm Sweden.

5. Department of Public Health & Caring Sciences, Uppsala University, Uppsala, Sweden.

6. Department of Public Health and Clinical Medicine, Family Medicine, Umeå University, Umeå, Sweden.

7. Department of Preventive Medicine and Public Health, Universidad Autónoma de Madrid, Madrid, Spain.

8. Department of Clinical Sciences in Malmö, Centre for Primary Health Care Research, Lund University, Lund, Sweden.

9. Centre of Medical Image Science and Visualization (CMIV), Linköping University, Linköping, Sweden.

10. Centre for Epidemiology and Community Medicine, Region Stockholm, Stockholm, Sweden.

11. The George Institute for Global Health, University of New South Wales, Sydney, Australia.

**Figures and Tables**

**Supplementary Figure 1.** Flow chart of the study.

**Supplementary Figure 2.** Distribution of the levels of self-rated health along Life’s Essential 8 categories by sex.

**Supplementary Figure 3**. Unadjusted and adjusted associations of Life’s Essential 8 component scores (per one unit SD) with poor self-rated health.

**Supplementary Figure 4.** Restricted cubic splines for the association of Life’s Simple 7 (0-7), Life’s Simple 7 (0-7) behavior and Life’s Simple 7 (0-7) factor scores with poor self-rated health.

**Supplementary Figure 5.** Restricted cubic splines for the association of Life’s Simple 7 (0-7), Life’s Simple 7 (0-7) behavior and Life’s Simple 7 (0-7) factor scores with poor mental health-related quality of life (upper row) and poor physical health-related quality of life (lower row).

**Supplementary Figure 6.** Restricted cubic splines for the association of Life’s Simple 7 (0-14), Life’s Simple 7 (0-14) behavior and Life’s Simple 7 (0-14) factor scores with poor self-rated health.

**Supplementary Figure 7.** Restricted cubic splines for the association of Life’s Simple 7 (0-14), Life’s Simple 7 (0-14) behavior and Life’s Simple 7 (0-14) factor scores with poor mental health-related quality of life (upper row) and poor physical health-related quality of life (lower row).

**Supplementary Figure 8.** Receiver operating characteristic curves of Life’s Essential 8, Life’s Simple 7 (0-7) and Life’s Simple 7 (0-14) to discriminate poor mental health-related quality of life and poor physical health-related quality of life.

**Supplementary Table 1**. Demographic and clinical characteristics of the included and excluded population.

**Supplementary Table 2.** Associations of Life’s Essential 8 and poor self-rated health.

**Supplementary Table 3.** Associations of Life’s Essential 8 and poor self-rated health, sensitivity analysis.

**Supplementary Table 4.** Associations of Life’s Essential 8 in relation to poor mental health-related quality of life and poor physical health-related quality of life .


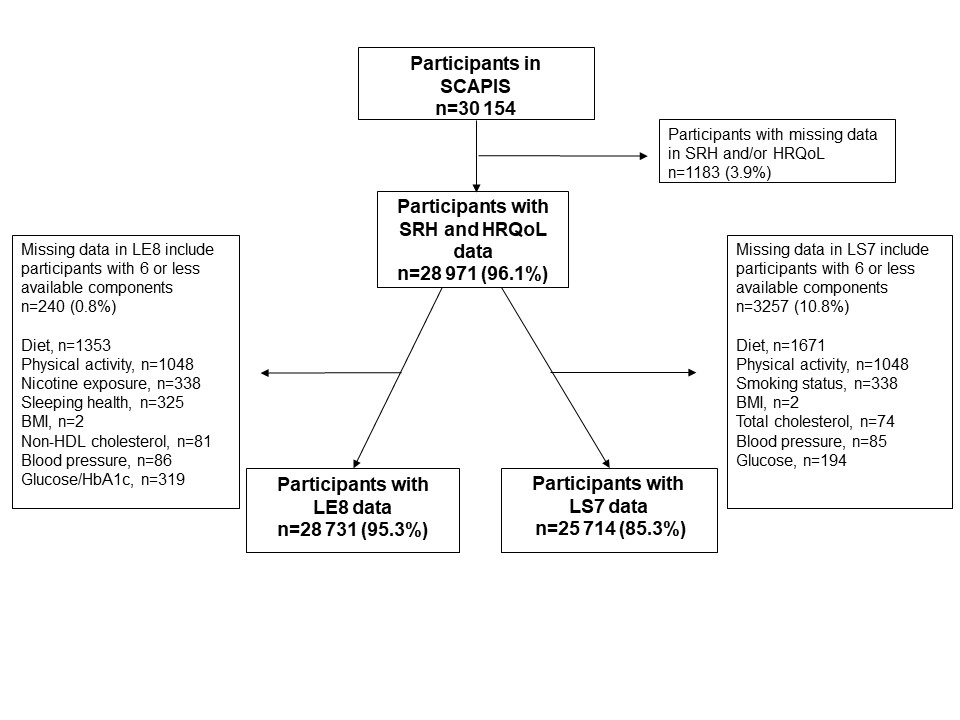


**Supplementary Figure 1. Flow chart of the study.**

BMI: body mass index, HbA1c: glycosylated hemoglobin, Non-HDL cholesterol: non-high density lipoprotein cholesterol, LE8: Life’s Essential 8, LS7: Life’s Simple 7, SCAPIS: Swedish CArdioPulmonary bioImage Study, SRH: self-rated health, HRQoL: health-related quality of life.

**Supplementary Figure 2. Distribution of the levels of self-rated health along Life’s Essential 8 categories by sex.**


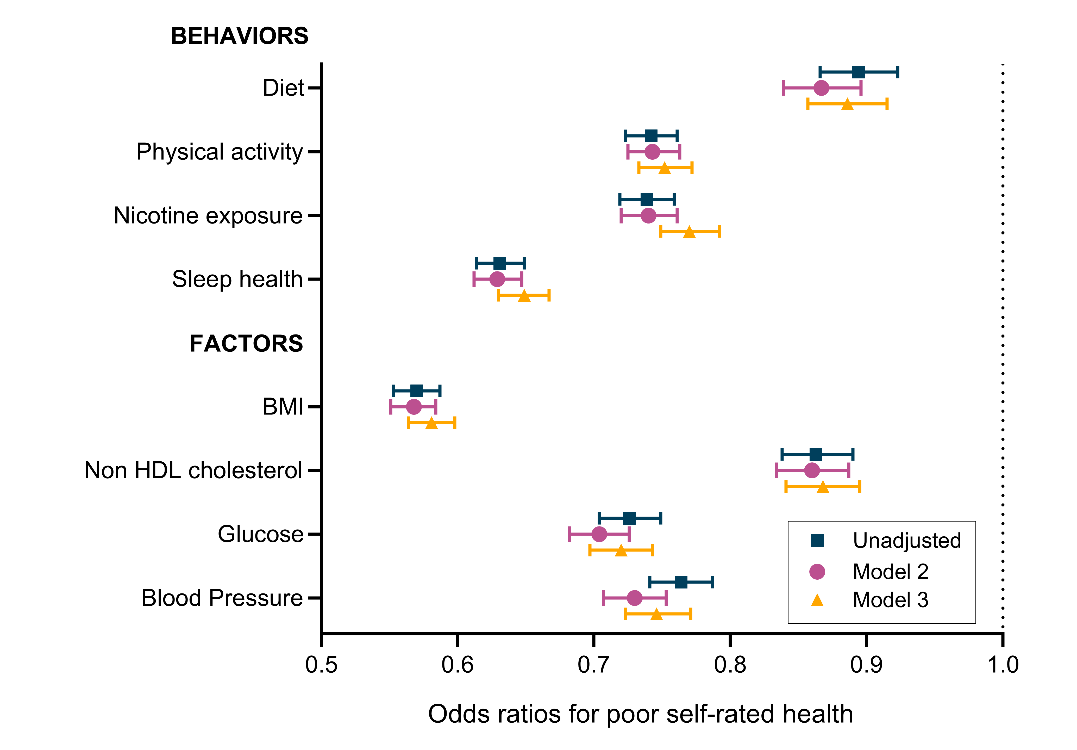


**Supplementary Figure 3. Unadjusted and adjusted associations of Life’s Essential 8 component scores (per one unit SD) with** **poor self-rated health.**

The association between LE8 component scores, as continuous 0-100 points values transformed to Z scores, and poor general self-rated health was modeled using binary logistic regression. Model 1, unadjusted; Model 2: adjusted by sex, age, and study site; Model 3: adjusted by Model 2 covariates + educational status, marital status, and chronic disease (myocardial infarction, stroke, heart failure, peripheral arterial disease, chronic obstructive pulmonary disease, celiac disease, Crohn’s disease or ulcerative colitis disease, rheumatic disease, and cancer).

BMI: body mass index, LE8: Life’s Essential 8, Non-HDL cholesterol: non-high density lipoprotein cholesterol, SD: standard deviation.


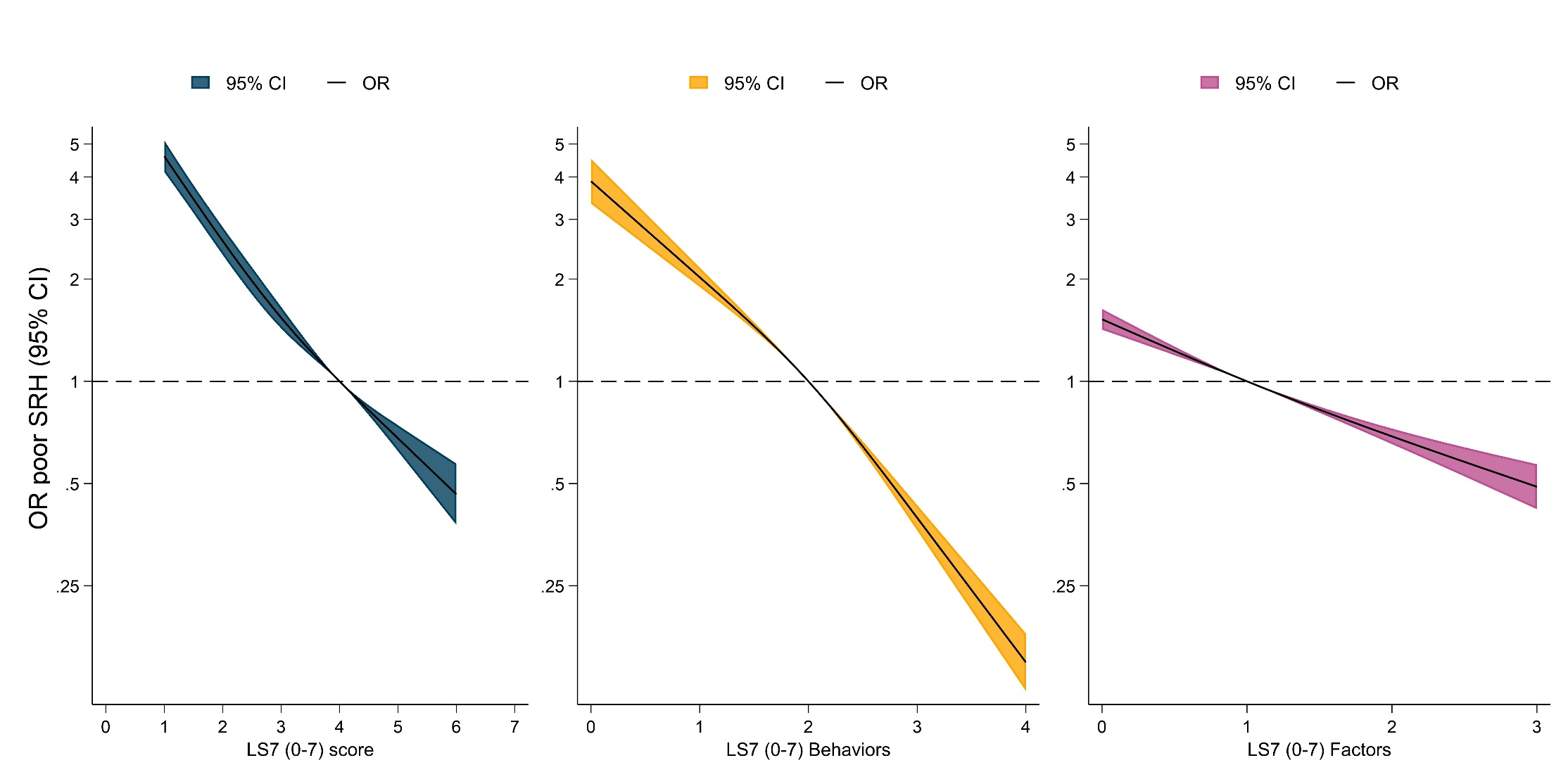


**Supplementary Figure 4. Restricted cubic splines for the association of Life’s Simple 7 (0-7), Life’s Simple 7 (0-7) behavior and Life’s Simple 7 (0-7) factor scores with poor self-rated health.**

All models are binary logistic regressions adjusted by age, sex, and site. X-axes were trimmed to depict the associations for the 1^st^ to 99^th^ percentile of LS7 (0-7) values. Reference points are settled at 4, 2 and 1 point for LS7 (0-7), LS7 (0-7) behavior and LS7 (0-7) factor scores, which represent the 81.7^th^, 68.3^th^ and 67.9^th^ percentiles, respectively.

CI: confidence interval, OR: odds ratio, LS7 (0-7): Life’s Simple 7 (scored as 0 to 7), SRH: self-rated health.


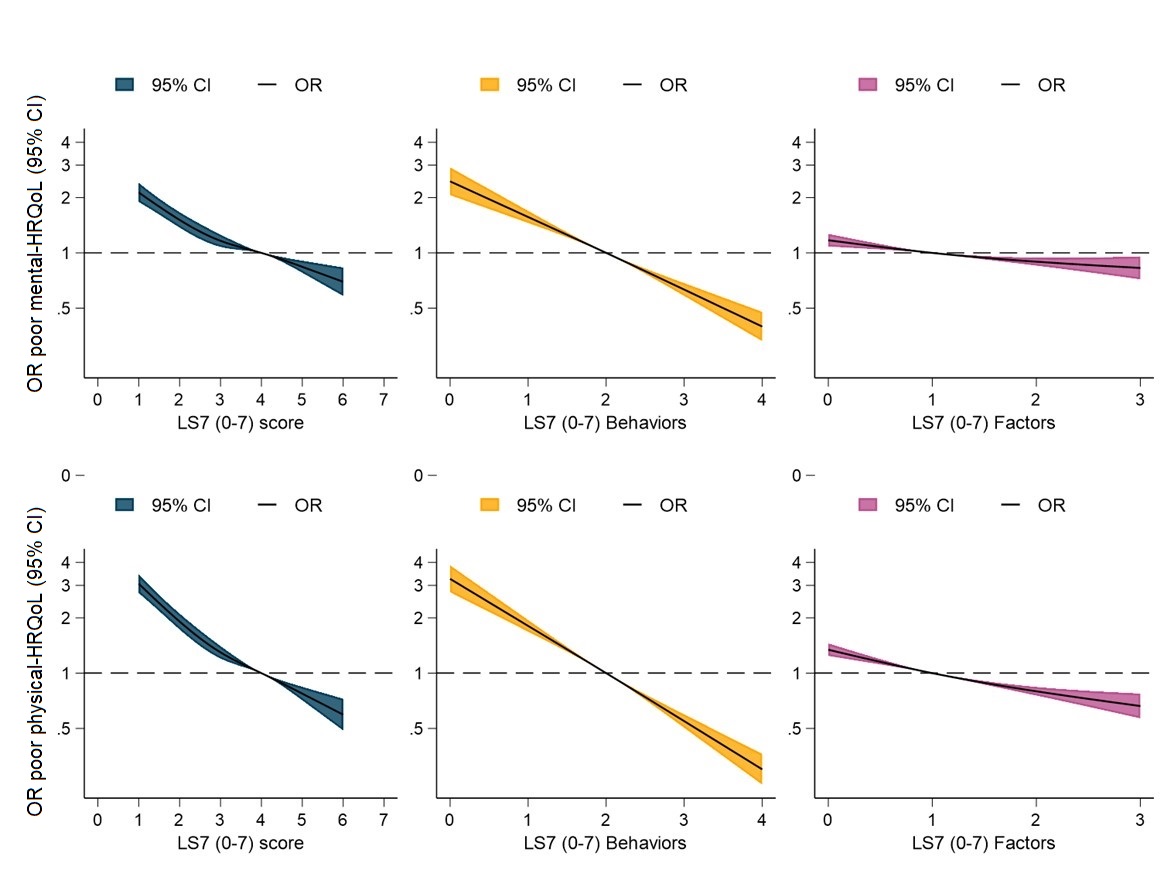


**Supplementary Figure 5. Restricted cubic splines for the association of Life’s Simple 7 (0-7), Life’s Simple 7 (0-7) behavior and Life’s Simple 7 (0-7) factor scores with poor mental health-related quality of life (upper row) and poor physical health-related quality of life (lower row).**

All models are binary logistic regressions adjusted by age, sex, and site. Reference points are settled at 4, 2 and 1 point for LS7 (0-7), LS7 (0-7) behavior and LS7 (0-7) factor scores, respectively. X-axes were trimmed to depict the associations for the 1^st^ to 99^th^ percentile of LS7 (0-7) values.

CI: confidence interval, OR: odds ratio, LS7 (0-7): Life’s Simple 7 (scored as 0 to 7), HRQoL: health-related quality of life.


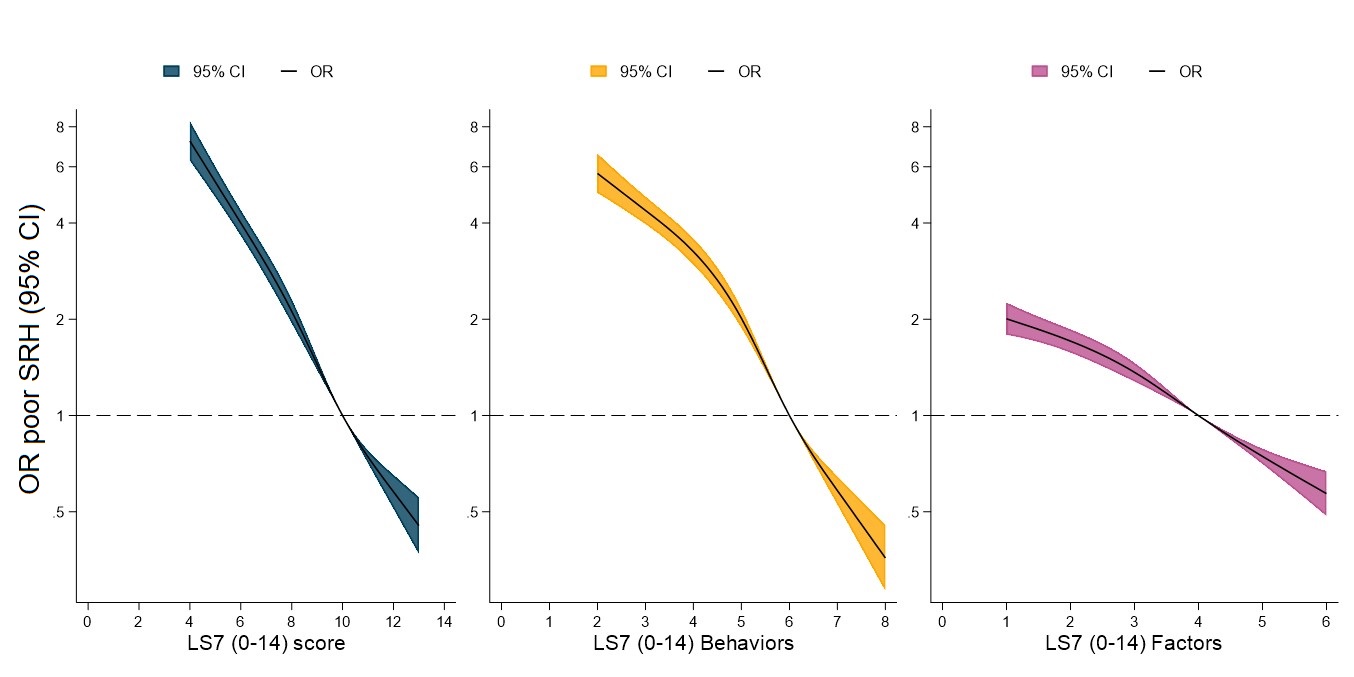


**Supplementary Figure 6. Restricted cubic splines for the association of Life’s Simple 7 (0-14), Life’s Simple 7 (0-14) behavior and Life’s Simple 7 (0-14) factor scores with poor self-rated health.**

All models are binary logistic regressions adjusted by age, sex, and site. X-axes were trimmed to depict the associations for the 1^st^ to 99^th^ percentile of LS7 (0-14) values. Reference points are settled at 10, 6 and 4 points, for LS7 (0-14) total, LS7 (0-14) behavior and LS7 (0-14) factor scores, which represent the 73.5^th^, 74.7^th^ and 75.2^th^ percentiles, respectively.

CI: confidence interval, OR: odds ratio, LS7 (0-14): Life’s Simple 7 (scored as 0 to 14), SRH: self-rated health.


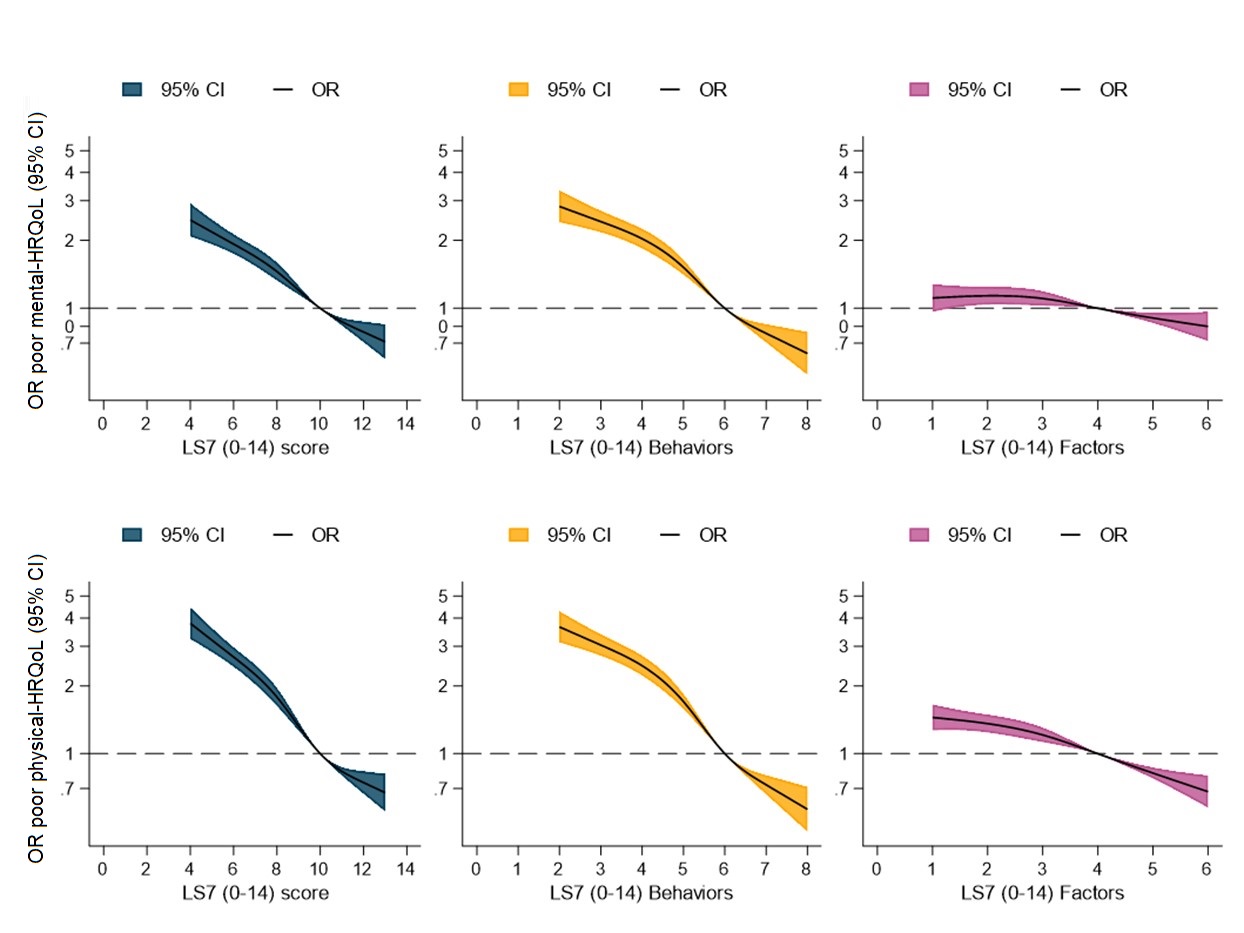


**Supplementary Figure 7. Restricted cubic splines for the association of Life’s Simple 7 (0-14), Life’s Simple 7 (0-14) behavior and Life’s Simple 7 (0-14) factor scores with poor mental health-related quality of life (upper row) and poor physical health-related quality of life (lower row).**

All models are binary logistic regressions adjusted by age, sex, and site. Reference points are settled at 10, 6 and 4 points, for the total, behavior and factor scores, respectively. X-axes were trimmed to depict the associations for the 1^st^ to 99^th^ percentile of LS7 (0-14) values. CI: confidence interval, OR: odds ratio, LS7 (0-14): Life’s Simple 7 (scored as 0 to 14), HRQoL: health-related quality of life.


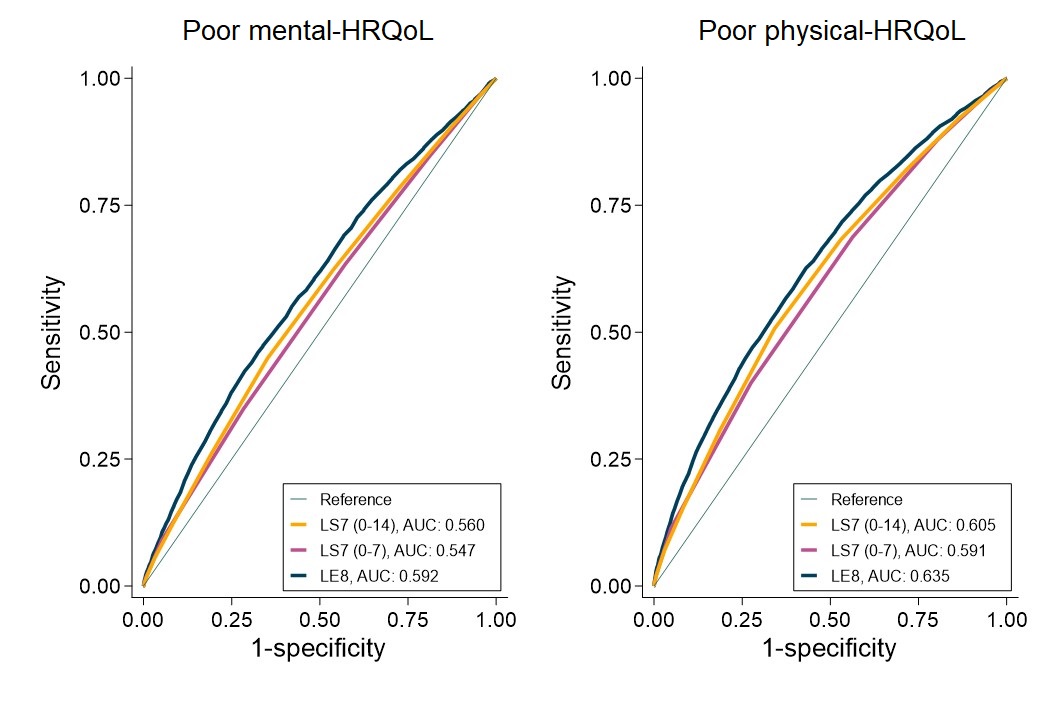


**Supplementary Figure 8. Receiver operating characteristic curves of Life’s Essential 8, Life’s Simple 7 (0-7) and Life’s Simple 7 (0-14) to discriminate poor mental health-related quality of life and poor physical health-related quality of life.**

AUC: area under curve, LE8: Life’s Essential 8 score., LS7 (0-7): Life’s Simple 7 (scored as 0 to 7), LS7 (0-14): Life’s Simple 7 (scored as 0 to 14), HRQoL: health-related quality of life.

| **Supplementary Table 1. Demographic and clinical characteristics of the included and excluded population.** | | |
| --- | --- | --- |
|  | **Included**  **n=28 731 (95.3%)** | **Excluded**  **n=1423 (4.7%)** |
| **Age and cardiovascular risk factors** |  |  |
| Age, y | 57.5 ± 4.3 | 57.6 ± 4.3 |
| BMI, kg/m^2^ | 26.9 ± 4.4 | 27.9 ± 5.1 |
| Obesity | 6082 (21.2) | 401 (28.3) |
| Total cholesterol, mg/dL | 212.2 ± 40.6 | 212.2 ± 43.0 |
| Hypercholesterolemia | 3342 (11.8) | 67 (13.3) |
| Systolic blood pressure, mmHg | 125.8 ± 17.0 | 127.3 ± 17.4 |
| Diastolic blood pressure, mmHg | 77.5 ± 10.5 | 77.9 ± 10.6 |
| Hypertension | 6450 (22.7) | 125 (24.8) |
| Fasting glucose, mg/dL | 103.2 ± 19.8 | 109.5 ± 31.2 |
| Glycosylated hemoglobin, mmol/mol | 36.5 ± 6.3 | 38.6 ± 9.2 |
| Diabetes mellitus | 1235 (4.4) | 53 (10.5) |
| Moderate-vigorous physical activity, min/week | 391.7 ± 208.4 | 372.3 ± 226.7 |
| LE8 diet (0-100) score | 41.1 ± 16.1 | 41.6 ± 16.7 |
| Smoking |  |  |
| Current | 3571 (12.6) | 257 (44.3) |
| Ex-smoker ≤1 year | 420 (1.5) | 11 (1.9) |
| Ex-smoker >1 year | 9962 (35.0) | 133 (22.9) |
| Never | 14484 (50.9) | 179 (30.9) |
| **Education level** |  |  |
| Unfinished primary school | 177 (0.6) | 18 (3.6) |
| Primary school | 2444 (8.5) | 86 (17.0) |
| Secondary school | 13 029 (45.5) | 242 (47.7) |
| University degree | 12 987 (45.4) | 161 (31.8) |
| **Current marital status** |  |  |
| Single | 3822 (13.4) | 120 (23.3) |
| Divorced | 3161 (11.1) | 89 (17.3) |
| Married | 21 107 (73.9) | 296 (57.5) |
| Widow | 475 (1.7) | 10 (1.9) |
| **Cardiovascular health scores** |  |  |
| LE8 (0-100) | 70.7 ± 11.6 | 61.1 ± 13.1 |
| LS7 (0-7) | 3.3 ± 1.3 | 2.5 ± 1.3 |
| LS7 (0-14) | 9.1 ± 2.0 | 7.6 ± 2.1 |
| **Self-rated health** |  |  |
| Poor | 5168 (18.0) | 93 (38.8) |
| **Health-related quality of life** |  |  |
| Poor mental | 4190 (15.1) | 60 (29.3) |
| Poor physical | 4159 (15.0) | 63 (30.7) |
| BMI: body mass index, LE8: Life’s Essential 8 score, LS7 (0-7): Life’s Simple 7 (scored as 0-7), LS7 (0-14): Life’s Simple 7 (scored as 0-14).  Data refer to mean ± standard deviation and frequencies (percentage). | | |

| **Supplementary Table 2. Associations of Life’s Essential 8 and poor self-rated health.** | | | | | | | | | | | | | | | | | | |
| --- | --- | --- | --- | --- | --- | --- | --- | --- | --- | --- | --- | --- | --- | --- | --- | --- | --- | --- |
|  | **Model 1** | | | | | | **Model 2** | | | | | | **Model 3** | | | | | |
|  | **LE8 total** | | **LE8 Behaviors** | | **LE8 Factors** | | **LE8 total** | | **LE8 Behaviors** | | **LE8 Factors** | | **LE8 total** | | **LE8 Behaviors** | | **LE8 Factors** | |
|  | **OR** | **95% CI** | **OR** | **95% CI** | **OR** | **95% CI** | **OR** | **95% CI** | **OR** | **95% CI** | **OR** | **95% CI** | **OR** | **95% CI** | **OR** | **95% CI** | **OR** | **95% CI** |
| **30** | 24.9 | (20.5-30.3)* | 8.4 | (7.2-9.9)* | 4.6 | (4.2-5.1) | 28.1 | (23.0-34.3)* | 8.5 | (7.2-10.0)* | 5.3 | (4.7-5.8) | 22.6 | (18.3-27.9)* | 6.1 | (5.1-7.2)* | 5.2 | (4.6-5.7) |
| **40** | 13.2 | (11.6-15.1)* | 5.9 | (5.3-6.6) | 3.3 | (3.1-3.6) | 14.8 | (13.0-17.0)* | 6.0 | (5.3-6.7) | 3.8 | (3.4-4.1) | 12.6 | (10.9-14.5)* | 4.5 | (4.0-5.1) | 3.7 | (3.4-4.1) |
| **50** | 7.0 | (6.4-7.7) | 4.1 | (3.8-4.5) | 2.4 | (2.2-2.6) | 7.8 | (7.2-8.6) | 4.2 | (3.8-4.6) | 2.7 | (2.4-2.9) | 7.0 | (6.3-7.7) | 3.4 | (3.1-3.7) | 2.7 | (2.4-2.9) |
| **60** | 3.7 | (3.4-4.0) | 2.8 | (2.6-3.1) | 1.8 | (1.6-1.9) | 4.1 | (3.7-4.5) | 2.9 | (2.6-3.1) | 1.9 | (1.8-2.1) | 3.8 | (3.5-4.2) | 2.5 | (2.3-2.7) | 1.9 | (1.8-2.1) |
| **70** | 1.8 | (1.7-1.9) | 1.8 | (1.7-1.9) | 1.3 | (1.3-1.4) | 2.0 | (1.9-2.1) | 1.8 | (1.7-1.9) | 1.4 | (1.3-1.4) | 1.9 | (1.8-2.0) | 1.7 | (1.6-1.8) | 1.4 | (1.3-1.4) |
| **80, ref.** | 1.0 | (1.0-1.0) | 1.0 | (1.0-1.0) | 1.0 | (1.0-1.0) | 1.0 | (1.0-1.0) | 1.0 | (1.0-1.0) | 1.0 | (1.0-1.0) | 1.0 | (1.0-1.0) | 1.0 | (1.0-1.0) | 1.0 | (1.0-1.0) |
| **90** | 0.7 | (0.6-0.7) | 0.8 | (0.7-0.9) | 0.8 | (0.7-0.8) | 0.6 | (0.5-0.7) | 0.8 | (0.7-0.9) | 0.8 | (0.7-0.8) | 0.6 | (0.5-0.7) | 0.8 | (0.7-0.9) | 0.8 | (0.7-0.8) |
| **100** | 0.4 | (0.3-0.6)* | 0.7 | (0.6-0.9)* | 0.6 | (0.5-0.7) | 0.4 | (0.3-0.5)* | 0.7 | (0.6-0.9)* | 0.6 | (0.5-0.7) | 0.4 | (0.3-0.6)* | 0.7 | (0.6-1.0)* | 0.6 | (0.5-0.7) |
| CI: confidence interval, LE8: Life’s Essential 8, OR: odds ratio, ref.: reference.  Models are computed as multivariate logistic restricted cubic splines. Model 1: unadjusted; Model 2: adjusted by age, sex, and site; Model 3: age, sex, site, educational status, marital status and chronic disease (myocardial infarction, stroke, heart failure, peripheral arterial disease, chronic obstructive pulmonary disease, celiac disease, Crohn’s disease or ulcerative colitis disease, rheumatic disease and cancer  In the corresponding figures depicting restricted cubic splines, X-axes were trimmed to depict the associations for the 1^st^ to 99^th^ percentile of LE8 values.  *These values are out of the trimmed values, which can render less consistent results. | | | | | | | | | | | | | | | | | | |

| **Supplementary Table 3. Associations of Life’s Essential 8 and poor self-rated health, sensitivity analysis.** | | | | | |
| --- | --- | --- | --- | --- | --- |
|  | **Reference analysis^1^** | |  | **Sensitivity analysis^2^** | |
|  | **LE8 total** | |  | **LE8 total** | |
|  | **N=28 731** | |  | **N=25 679** | |
|  | **OR** | **95% CI** |  | **OR** | **95% CI** |
| **30** | 28.1 | (23.0-34.3) |  | 28.3 | (22.8-35.2) |
| **40** | 14.8 | (13.0-17.0) |  | 15.1 | (13.0-17.5) |
| **50** | 7.8 | (7.2-8.6) |  | 8.1 | (7.3-8.9) |
| **60** | 4.1 | (3.7-4.5) |  | 4.3 | (3.9-4.7) |
| **70** | 2.0 | (1.9-2.1) |  | 2.1 | (1.9-2.2) |
| **80, ref.** | 1.0 | (1.0-1.0) |  | 1.0 | (1.0-1.0) |
| **90** | 0.6 | (0.5-0.7) |  | 0.6 | (0.5-0.7) |
| **100** | 0.4 | (0.3-0.5) |  | 0.3 | (0.2-0.5) |
| CI: confidence interval, LE8: Life’s Essential 8, OR: odds ratio, ref.: reference.  Models are computed as multivariate logistic restricted cubic splines considering Model 2: adjusted by age, sex, and site.  1. Reference analysis: LE8 total is computed considering ≥7 components.  2. Sensitivity analysis: LE8 total is computed considering 8 components. | | | | | |

| **Supplementary Table 4. Associations of Life’s Essential 8 and poor mental health-related quality of life and poor physical health-related quality of life.** | | | | | | | | | | | | | | | | | | |
| --- | --- | --- | --- | --- | --- | --- | --- | --- | --- | --- | --- | --- | --- | --- | --- | --- | --- | --- |
| **Model 1** | | | | | | | **Model 2** | | | | | | **Model 3** | | | | | |
|  | **LE8 total** | | **LE8 Behaviors** | | **LE8 Factors** | | **LE8 total** | | **LE8 Behaviors** | | **LE8 Factors** | | **LE8 total** | | **LE8 Behaviors** | | **LE8 Factors** | |
|  | **OR** | **95% CI** | **OR** | **95% CI** | **OR** | **95% CI** | **OR** | **95% CI** | **OR** | **95% CI** | **OR** | **95% CI** | **OR** | **95% CI** | **OR** | **95% CI** | **OR** | **95% CI** |
| **Mental health-related quality of life** | | | | | | | | | | | | | | | | | | |
| **30** | 5.9 | (4.7-7.3)* | 5.5 | (4.6-6.5)* | 1.6 | (1.4-1.8) | 7.3 | (5.8-9.1)* | 5.4 | (4.5-6.5)* | 1.9 | (1.7-2.2) | 6.0 | (4.8-7.6)* | 4.2 | (3.5-5.1)* | 1.8 | (1.6-2.1) |
| **40** | 4.0 | (3.4-4.6)* | 4.2 | (3.7-4.7) | 1.5 | (1.3-1.6) | 4.9 | (4.2-5.6)* | 4.2 | (3.7-4.8) | 1.8 | (1.6-1.9) | 4.2 | (3.6-4.9)* | 3.4 | (3.0-3.9) | 1.7 | (1.6-1.9) |
| **50** | 2.7 | (2.4-2.9) | 3.2 | (2.9-3.5) | 1.3 | (1.2-1.5) | 3.3 | (3.0-3.6) | 3.2 | (2.9-3.5) | 1.6 | (1.4-1.7) | 2.9 | (2.6-3.2) | 2.8 | (2.5-3.0) | 1.6 | (1.4-1.7) |
| **60** | 1.8 | (1.6-2.0) | 2.4 | (2.2-2.6) | 1.2 | (1.1-1.3) | 2.2 | (2.0-2.4) | 2.4 | (2.2-2.7) | 1.4 | (1.3-1.5) | 2.0 | (1.8-2.2) | 2.2 | (2.0-2.4) | 1.4 | (1.3-1.5) |
| **70** | 1.3 | (1.2-1.3) | 1.6 | (1.5-1.8) | 1.1 | (1.0-1.1) | 1.4 | (1.4-1.5) | 1.7 | (1.6-1.8) | 1.2 | (1.1-1.2) | 1.4 | (1.3-1.5) | 1.6 | (1.5-1.7) | 1.2 | (1.1-1.2) |
| **80, ref.** | 1.0 | (1.0-1.0) | 1.0 | (1.0-1.0) | 1.0 | (1.0-1.0) | 1.0 | (1.0-1.0) | 1.0 | (1.0-1.0) | 1.0 | (1.0-1.0) | 1.0 | (1.0-1.0) | 1.0 | (1.0-1.0) | 1.0 | (1.0-1.0) |
| **90** | 0.9 | (0.8-1.0) | 0.9 | (0.8-1.0) | 1.0 | (0.9-1.1) | 0.7 | (0.6-0.8) | 0.8 | (0.7-0.9) | 0.9 | (0.9-1.0) | 0.7 | (0.7-0.8) | 0.8 | (0.7-0.9) | 0.9 | (0.9-1.0) |
| **100** | 0.7 | (0.6-1.0)* | 0.8 | (0.6-1.1)* | 1.0 | (0.9-1.2) | 0.5 | (0.4-0.7)* | 0.7 | (0.5-0.9)* | 0.8 | (0.7-1.0) | 0.6 | (0.4-0.7)* | 0.7 | (0.5-0.9)* | 0.9 | (0.8-1.0) |
| **Physical health-related quality of life** | | | | | | | | | | | | | | | | | | |
| **30** | 11.5 | (9.3-14.2)* | 6.0 | (5.0-7.1)* | 2.7 | (2.4-3.0) | 13.9 | (11.3-17.3)* | 6.0 | (5.0-7.2)* | 3.2 | (2.9-3.6) | 11.0 | (8.8-13.7)* | 4.5 | (3.7-5.4)* | 3.0 | (2.6-3.3) |
| **40** | 6.7 | (5.8-7.7)* | 4.3 | (3.8-4.9) | 2.2 | (2.0-2.4) | 8.0 | (7.0-9.3)* | 4.4 | (3.9-5.0) | 2.5 | (2.3-2.8) | 6.6 | (5.7-7.7)* | 3.5 | (3.0-4.0) | 2.4 | (2.2-2.7) |
| **50** | 3.9 | (3.6-4.3) | 3.2 | (2.9-3.5) | 1.7 | (1.6-1.9) | 4.6 | (4.2-5.1) | 3.2 | (2.9-3.5) | 2.0 | (1.8-2.2) | 4.0 | (3.6-4.5) | 2.7 | (2.4-2.9) | 2.0 | (1.8-2.2) |
| **60** | 2.3 | (2.1-2.5) | 2.3 | (2.1-2.5) | 1.4 | (1.3-1.5) | 2.7 | (2.4-2.9) | 2.3 | (2.1-2.5) | 1.6 | (1.5-1.7) | 2.4 | (2.2-2.7) | 2.0 | (1.8-2.2) | 1.6 | (1.4-1.7) |
| **70** | 1.4 | (1.4-1.5) | 1.6 | (1.4-1.7) | 1.2 | (1.1-1.2) | 1.6 | (1.5-1.7) | 1.6 | (1.5-1.7) | 1.3 | (1.2-1.3) | 1.5 | (1.4-1.6) | 1.5 | (1.4-1.6) | 1.2 | (1.2-1.3) |
| **80, ref.** | 1.0 | (1.0-1.0) | 1.0 | (1.0-1.0) | 1.0 | (1.0-1.0) | 1.0 | (1.0-1.0) | 1.0 | (1.0-1.0) | 1.0 | (1.0-1.0) | 1.0 | (1.0-1.0) | 1.0 | (1.0-1.0) | 1.0 | (1.0-1.0) |
| **90** | 0.8 | (0.7-0.9) | 0.9 | (0.8-0.9) | 0.9 | (0.8-0.9) | 0.7 | (0.6-0.8) | 0.8 | (0.7-0.9) | 0.8 | (0.8-0.9) | 0.7 | (0.6-0.8) | 0.8 | (0.7-0.9) | 0.8 | (0.8-0.9) |
| **100** | 0.6 | (0.4-0.8)* | 0.8 | (0.6-1.0)* | 0.7 | (0.6-0.9) | 0.5 | (0.4-0.6)* | 0.7 | (0.6-0.9)* | 0.7 | (0.6-0.8) | 0.5 | (0.4-0.7)* | 0.7 | (0.6-1.0)* | 0.7 | (0.6-0.8) |
| CI: confidence interval, LE8: Life’s Essential 8, OR: odds ratio, ref.: reference.  Models are computed as multivariate logistic restricted cubic splines. Model 1: unadjusted; Model 2: adjusted by age, sex, and site; Model 3: age, sex, site, educational status, marital status and chronic disease (myocardial infarction, stroke, heart failure, peripheral arterial disease, chronic obstructive pulmonary disease, celiac disease, Crohn’s disease or ulcerative colitis disease, rheumatic disease and cancer  In the corresponding figures depicting restricted cubic splines, X-axes were trimmed to depict the associations for the 1^st^ to 99^th^ percentile of LE8 values.  *These values are out of the trimmed values, which can render less consistent results. | | | | | | | | | | | | | | | | | | |
